# Mitochondrial fusion by pharmacological manipulation impedes somatic cell reprogramming to pluripotency: New insight into the role of mitophagy in cell stemness

**DOI:** 10.18632/aging.100465

**Published:** 2012-06-16

**Authors:** Alejandro Vazquez-Martin, Sílvia Cufí, Bruna Corominas-Faja, Cristina Oliveras-Ferraros, Luciano Vellon, Javier A. Menendez

**Affiliations:** ^1^ Translational Research Laboratory, Catalan Institute of Oncology (ICO), Girona, Spain; ^2^ Girona Biomedical Research Institute, Girona (IDIBGi), Spain; ^3^ Reprogramming Unit, Fundación INBIOMED, San Sebastián, Gipuzkua, Spain

**Keywords:** mitochondrial division regulates somatic cell reprogramming

## Abstract

Recent studies have suggested a pivotal role for autophagy in stem cell maintenance and differentiation. Reprogramming of somatic cells to induced pluripotent stem cells (iPSCs) has been also suggested to bio-energetically take advantage of mitochondrial autophagy (mitophagy). We have preliminary addressed how mitophagy might play a role in the regulation of induced pluripotency using mdivi-1 (for mitochondrial division inhibitor), a highly efficacious small molecule that selectively inhibits the self-assembly of DRP1, a member of the dynamin family of large GTPases that mediates mitochondrial fission. At mdivi-1 concentrations that rapidly induced the formation of mitochondrial net-like or collapsed perinuclear mitochondrial structures, we observed that the reprogramming efficiency of mouse embryonic fibroblasts transduced with the Yamanaka three-factor cocktail (OCT4, KLF4, and SOX2) is drastically reduced by more than 95%. Treatment of MEFs with mdivi-1 at the early stages of reprogramming before the appearance of iPSC colonies was sufficient to completely inhibit somatic cell reprogramming. Therefore, the observed effects on reprogramming efficiencies were due likely to the inhibition of the process of reprogramming itself and not to an impairment of iPSC colony survival or growth. Moreover, the typical morphology of established iPSC colonies with positive alkaline phosphatase staining was negatively affected by mdivi-1 exposure. In the presence of mdivi-1, the colony morphology of the iPSCs was lost, and they somewhat resembled fibroblasts. The alkaline phosphatase staining was also significantly reduced, a finding that is indicative of differentiation. Our current findings provide new insight into how mitochondrial division is integrated into the reprogramming factors-driven transcriptional network that specifies the unique pluripotency of stem cells.

Although the roles of autophagy in the biology of stem cells have just started to be explored, we are beginning to accumulate strong evidence suggesting that a catabolic process whereby cells generate energy and building blocks by promoting large-scale recycling of cytoplasmic macromolecules and organelles including mitochondria may be essential for the acquisition, maintenance, and exit of stem cell-defining self-renewing pluripotent states. Di Gioacchino and colleagues [1] were pioneers at demonstrating that an autophagic phenotype could mitigate metal-induced toxicity in stem and progenitor cells, thus contributing to the conservation of tissue renewal capacity. Liu and colleagues [2] demonstrated for the first time that a protein playing an important role in autophagy (FIP200, focal adhesion kinase family interacting protein of 200 kDa) was cell-autonomously required for the maintenance and function of fetal hematopoietic stem cells (HSCs). Of note, FIP200-null fetal HSCs displayed both increased mitochondrial mass and increased reactive oxygen species (ROS) [2, 3]. Without studying the link to autophagy *per se*, three accompanying papers published in the same volume of *Nature* underlined the critical importance of coupling energy metabolism and stem-cell homeostasis [4-6]. The three groups revealed that loss of the metabolic sensor *LKB1/STK1*, a tumor-suppressor protein that induces autophagy (its loss reduces autophagy) [7, 8] causes a loss of HSC quiescence followed by a rapid depletion of all hematopoietic subpopulations. The deleterious hematopoietic effects triggered by LKB1 inactivation were accompanied by depletion of cellular ATP and mitochondrial defects [4-6]. Mortensen and colleagues [9] unambiguously demonstrated that the selective removal of mitochondria, but not other organelles, by autophagy (mitophagy) is a necessary developmental step in erythroid cells. Using mice lacking the essential autophagy gene ATG7 in the hematopoietic system, which develop severe anemia and lymphopenia, ATG7^−/−^ erythrocytes and mature T lymphocytes were found to accumulate damaged mitochondria with altered membrane potential, ultimately leading to cell death [9]. When disabling autophagy in HSCs by conditionally deleting ATG7 in the hematopoietic system, the same authors observed that the hematopoietic stem and progenitor cell compartment displayed an accumulation of mitochondria and ROS as well as increased proliferation and DNA damage [10]. Indeed, the loss of autophagy in HSCs leads to the expansion of a progenitor cell population in the bone marrow that exhibits invasive myeloproliferation, thus resembling human acute myeloid leukemia (AML) [10, 11]. Mitophagy, therefore, seems to be a pivotal mechanism that protects HSCs from cellular damage and is essential to prevent hematopoietic malignancies.

## Bioenergetic transitions into pluripotency: A role for mitophagy

Because stem cells need to protect their genome from damage to maintain both the progenitor pool and their self-renewal capacity [12] and because intracellular ROS levels influence the long-term self-renewal capacity of HSCs [13-15], the above-mentioned studies strongly suggest that mitophagy protects the genome due to its ability to clear mitochondria as a source of ROS; therefore, mitophagy may help stem cells to maintain their self-renewal and pluripotent capacities [16-18]. However, it remains to be elucidated whether mitophagy is mechanistically linked to the acquisition of pluripotency. Recent studies have demonstrated that undifferentiated pluripotent stem cells display lower levels of mitochondrial mass and oxidative phosphorylation and that they preferentially use non-oxidative glycolysis as a major source of energy. Folmes and colleagues [19] confirmed that the stemness factor-mediated reprogramming of somatic cells into induced pluripotent stem cells (iPSCs) remarkably reverts mitochondrial networks into cristae-poor structures. Second, as has been previously shown by Prigione & Adjaye [20], the functional metamorphosis of somatic oxidative phosphorylation into acquired pluripotent glycolytic metabolism corresponds to an embryonic-like original pattern [19]. Thus, somatic mitochondria within human iPSCs suffer a reversion to an immature embryonic stem cell (ESC)-like state with respect to organelle morphology, distribution, and function, suggesting that the mitochondrial/oxidative stress pathway is actively modulated during cellular reprogramming to induce a rejuvenated state capable of escaping cellular senescence [21]. Indeed, Folmes's metaboproteomic studies demonstrated that cell fate during reprogramming is determined by the upregulation of glycolytic enzymes and the downregulation of electron transport chain complex I subunits. Temporal sampling demonstrated glycolytic gene potentiation prior to the induction of pluripotent markers; accordingly, stimulating glycolysis promotes reprogramming, and inhibiting glycolytic enzyme activity blunts reprogramming efficiency [19, 22]. Panopoulos and colleagues [23] have recently confirmed that a bioenergetic shift from somatic oxidative mitochondria toward an alternative ATP-generating glycolytic phenotype maximizes the efficiency of somatic reprogramming to pluripotency. In their hands, somatic cells that demonstrated oxidative:glycolytic energy production ratios closer to pluripotent cells reprogrammed more quickly and efficiently. Altogether, these studies strongly suggest that changes in metabolism may play a role in enabling the reprogramming process to occur rather than simply being a consequence of acquiring a pluripotent state.

Because the *a priori* energetic infrastructure of somatic cells appears to be a crucial molecular feature for achieving an optimal routing to pluripotency, it is tempting to suggest that beyond the importance of mitophagy in the turnover of dysfunctional mitochondria, it may also facilitate the metabolic switch from mitochondrial respiration to glycolysis that appears to underlie the acquisition of induced pluripotency [24]. The hypothesis that regulation of mitochondrial dynamics can specifically segregate the mitochondria that are destined for clearance through mitophagy is attractive because this process should result in compartmentalized cellular catabolism, loss of mitochondrial function, increased glucose uptake and, consequently, accelerated onset of pro-reprogramming glycolysis. On the one hand, recent studies have revealed that autophagy facilitates glycolysis during Ras-mediated oncogenic transformation [25]. Similar to its behavior during the reprogramming of somatic cells to iPSCs, mitochondrial respiration in cells engineered to overexpress Ras significantly declines in parallel with the acquisition of transformation characteristics [26]. The decreased respiration was not related to mitochondrial biogenesis, but it was inversely associated with the increased formation of autophagic acidic vesicles enclosing the mitochondria (mitophagy). On the other hand, Chen and colleagues [27, 28] recently demonstrated that rapamycin or PP242, two well-recognized pharmacological inducers of autophagy via inhibition of the mammalian target of rapamycin (mTOR) pathway, notably enhance the efficiency of reprogramming somatic cells to iPSCs. Moreover, treatment with mTOR inhibitors does not compromise the pluripotency of iPSCs. It is plausible that an mTOR-regulated increase in mitochondrial fission during the reprogramming of somatic cells to iPSCs may upregulate mitophagy, which could therefore lead to a significant reduction in both the number and the size of mitochondria to achieve the “mitochondrial phenotype” that is associated with stem cells. Conversely, an increase in mitochondrial fusion during the reprogramming of somatic cells might downregulate mitophagy, thus generating giant mitochondria that are associated with cell senescence, which is a pivotal roadblock during the reprogramming process when generating iPSCs. Accordingly, recent studies in our own laboratory have confirmed that the AMPK agonist metformin, which has been shown to promote a striking enlargement of mitochondria [29], efficiently impedes reprogramming of somatic cells to iPSCs [30]. However, none of the above-mentioned studies has definitively established a *bona fide* causal linkage between mitochondrial division and mitophagy with the acquisition of stem cell-like properties.

We recently addressed whether mitophagy might play a role in the regulation of induced pluripotency using mdivi-1 (for mitochondrial division inhibitor), a highly efficacious small molecule that selectively inhibits the self-assembly of DRP1 [31-33], a member of the dynamin family of large GTPases that mediates mitochondrial fission [34-36]. We now provide the first experimental evidence that mitochondrial division is integrated into the reprogramming factors-driven transcriptional network that specifies the unique pluripotency of stem cells.

## Pharmacological inhibition of DRP1 efficiently promotes mitochondrial fusion

**Figure 1:**
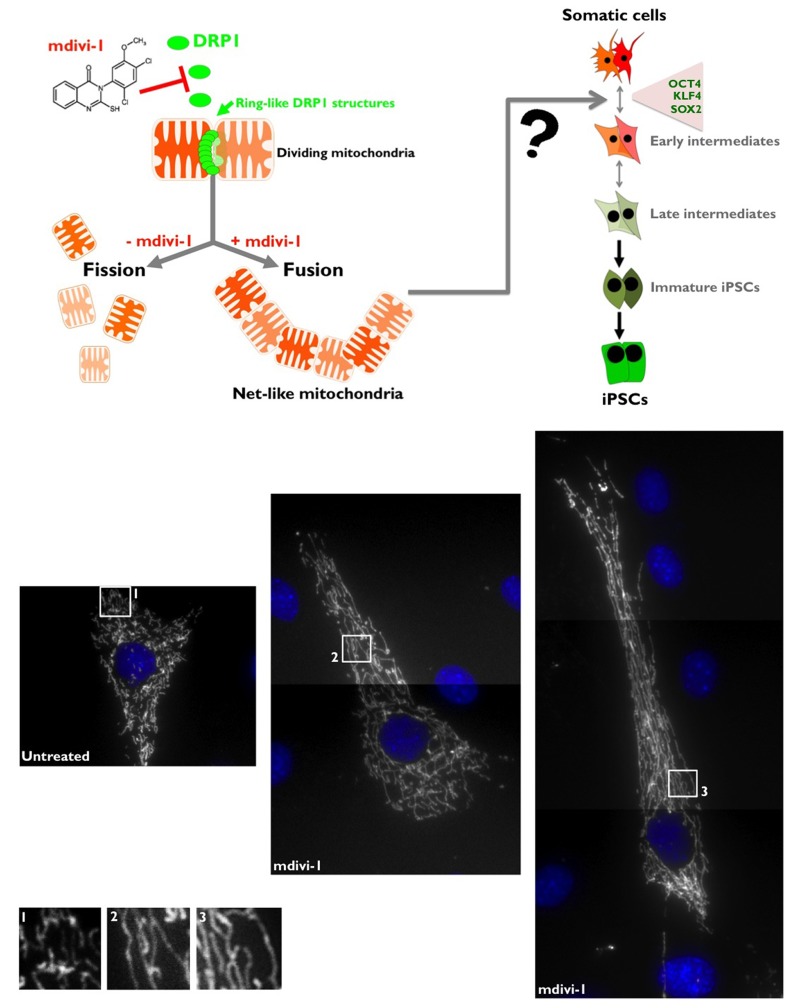
*Top.* mdivi-1 blocks the machinery of mitochondrial fission Mitochondria fission is crucially regulated by the activity of DRP1, which has a sequence homology with the GTPases dynamins that regulate vesicular trafficking and endocytosis. Although the exact molecular mechanism of DRP1 in the process of mitochondrial fission is still subject of debate, one of the well-accepted models is that DRP1 acts as a mechanoenzyme that self-assemble into spirals and onto lipid bilayers forming DRP1 decorated lipid tubes that undergo a large conformational change upon GTP addition resulting in membrane constriction and, therefore, mitochondrial division. DRP1 is a protein that is mainly distributed in the cytoplasm, but there is a fraction that localizes to specific points of the external mitochondrial membrane; these points mark the fission sites in dividing mitochondria. mdivi-1 [3-(2,4-Dichloro-5-methoxy-phenyl)-2-thioxo-1H-quinazolin-4-one] is a cell-permeable quinazolinone compound that inhibits DRP1 and effectively induces mitochondrial fusion into net-like structures (IC50 = 50 μmmol/L in COS cultures) in a reversible manner. mdivi-1 blocks DRP1 GTPase activity and self-assembly by an allosteric modulation-based mechanism. ***Bottom.* mdivi-1 inhibits mitochondrial division in MEFs.** MEFs were labeled with DsRed-Mito for the visualization of mitochondrial morphology. Untreated control cells showed a relatively tubular morphology that is maintained because mitochondrial fission and fusion occur in a balanced frequency, in contrast to the extremely long nets of interconnected mitochondria that collapse and aggregate after treatment with the DRP1 inhibitor mdivi-1.

Two distinct dynamin-related GTPases (DRPs), which function via self-assembly to regulate membrane dynamics in a variety of cellular events, are required for mitochondrial fusion [39, 40]. MFN1/2/Fzo1 (human/yeast nomenclature) and OPA1/Mgm1 drive outer and inner mitochondrial membrane fusion, respectively. A single DRP, DRP1/Dnm1, is required for mitochondrial fission [40, 41]. DRP1 is assembled from the cytosol onto mitochondria at focal sites of division [34], forming spiral chains around membrane constriction sites [35]. DRP1 self-assembly facilitates GTP hydrolysis and thereby organelle fission. In mammalian cells, when mitochondrial division is retarded by the expression of dominant-negative DRP1 or by RNAi of mitochondrial division proteins, tubular mitochondria become progressively more interconnected to form net-like structures and also collapse into degenerate perinuclear structures. However, overexpression of wild-type DRP1 does not lead to mitochondria fragmentation, suggesting that a simple alteration of DRP1 levels could not alter mitochondrial fission. Regulation of DRP1 properties, such as mitochondrial translocation, higher order assembly or GTPase activity, is critical [36, 39]. Here, we used a small molecular inhibitor of DRP1 to probe the mechanistic role that mitochondrial division plays in both the acquisition and the maintenance of pluripotency. We employed mdivi-1 (for mitochondrial division inhibitor), an inhibitor of mitochondrial division identified by Cassidy-Stone and colleagues using yeast screens of chemical libraries [31]. Because it is thought that mitochondrial fission is related to the progression of mitophagy, the inhibition of mitochondrial fission by mdivi-1, a specific inhibitor of DRP1-GTPase, has been shown to compromise mitophagy [31]. The addition of mdivi-1 to mammalian cells in culture has been shown to cause a rapid and reversible formation of mitochondrial net-like and degenerate perinuclear structures, consistent with attenuation in mitochondrial division [31, 33]. Indeed, depletion of DRP1 by RNAi causes the formation of net-like or collapsed perinuclear mitochondrial structures in mammalian cells, and treatment of these cells with mdivi-1 does not produce any additional changes to mitochondrial morphology, thus substantiating that DRP1 is the specific target of mdivi-1 in mammalian cells [31]. Our first step in determining the function DRP1-regulated mitochondrial dynamics was to confirm the effects of mdivi-1 on mitochondrial morphology. Using DsRed-Mito to label mitochondria, control MEFs displayed primary tubular and long mitochondria (Fig. 1, *top*). mdivi-1 treatment caused the formation of net-like mitochondria, as expected from its ability to directly attenuate mitochondrial division (Fig. 1, *bottom*).

## The DRP1-GTPase inhibitor mdivi-1 impedes reprogramming of human fibroblasts to iPSCs

To address the functional effects of a mitochondrial fission deficit imposed by DRP1-GTPase inhibition on iPSC generation, we performed comparison experiments using the three-factor (i.e., OCT4, SOX2, and KLF4) induction protocol in early-passage mouse embryonic fibroblasts (MEFs). MEFs were first transduced with individual lentiviruses containing OCT4, SOX2, and KLF4 at a 1:1:1 ratio on day 0. The transduction was repeated every 12 h for 2 days using the same batch of all three lentiviruses. On day three after the first transduction, the culture medium was switched to human embryonic stem (hES) cell growth medium with or without two different concentrations of mdivi-1. We used mdivi-1 at 10 and 50 μmol/L, the range of mdivi-1 concentrations required to observe either net-like or collapsed/degenerate perinuclear mitochondrial structures [31, Fig. 1, *bottom*]; the ES medium with or without mdivi-1 was renewed every two days. From days 10-12, clearly recognizable, tightly packed colonies similar to hES cells appeared in the mdivi-1-free control cultures. We then combined the observations of ES cell-like morphological changes (e.g., defined boundaries and high nucleus-to-cytoplasm ratio within individual cells) with alkaline phosphatase (AP) staining, a commonly used pluripotency marker, to quantify bona fide iPSC colonies on day 14 post-viral transduction.

**Figure 2:**
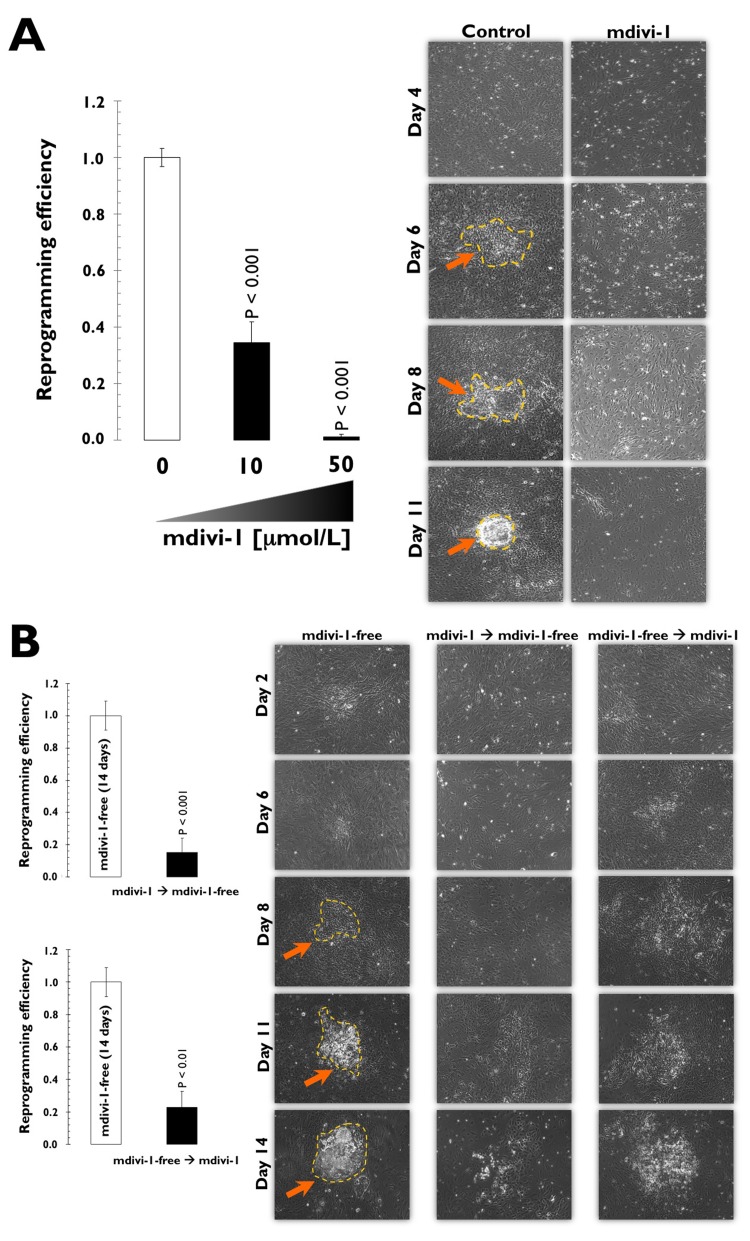
(A) Mouse embryonic fibroblasts (MEFs) fail to reprogram into induced pluripotent stem cells (iPSCs) in the presence of the DRP1 inhibitor mdivi-1 *Left.* Early passage MEFs infected with retroviruses encoding OCT4, SOX2, and KLF4 (OSK) were cultured in ES medium in the continuous presence or absence of mdivi-1 (10 and 50 μmol/L), as specified. *Top.* The numbers of AP+ colonies were counted 14 days after the initial infection and were plotted for each condition relative to the controls (x-fold), as specified. The error bars indicate the SEM. *Right.* Phase-contrast microphotographs of representative MEFs transduced with OSK at different time-points during the reprogramming process in the absence or presence of continuous mdivi-1 (50 μmol/L). The arrows indicate emerging iPSC-like colonies. (**B**) **DRP1 inactivation impedes early stem cell genetic reprogram-ming.** The early passage MEFs infected with retroviruses encoding the OSK stemness factors were grown in ES medium in the intermittent presence or absence of mdivi-1 (50 μmol/L), as specified. *Left.* The numbers of AP+ colonies were counted 14 days after the initial infection and are plotted for each condition relative to the controls (x-fold), as specified. The error bars indicate the SEM. *Right.* Phase-contrast microphotographs of representative MEFs transduced with OSK at different time-points during the reprogramming process in the absence or presence of intermittent mdivi-1 (50 μmol/L), as specified. Arrows indicate emerging iPSC-like colonies.

When MEFs were transduced with the three stemness factors in the absence of mdivi-1, we consistently obtained ~100 colonies from 50,000 starting cells (Fig. 2). Using the same rigorous criteria for calculating reprogramming efficiency, 30 AP+ colonies from 50,000 starting cells (~70% decrease) were generated in the reprogramming experiments that were performed in the presence of 10 μmol/L mdivi-1 (Fig. 2). Notably, only one to two colonies (more than a 95% decrease) were observed in parallel experiments when the mdivi-1 concentration was increased to 50 μmol/L (Fig. 2A). The mdivi-1-induced reduction in reprogramming efficiency we observed was independent of mdivi-1-induced cell death of the starting somatic population (Fig. 2). Whereas reprogrammed MEFs displayed an undifferentiated phenotype with distinct ES-like colonies in the absence of mdivi-1, flattened fibroblast-like cells with a low nucleus-to-cytoplasm ratio within individual cells were found when reprogramming was performed in the presence of mdivi-1 (Fig. 2A). To further confirm that the impaired reprogramming efficiency was not due to the mdivi-1-induced inhibition of established iPSC colonies, we treated MEFs with mdivi-1 either at early stages (days one to seven post-viral transduction) or at later stages of reprogramming (days seven to 14 post-viral transduction) but before colony appearance (day 10) (Fig. 2B). We found that treatment with mdivi-1 during the early stages of reprogramming notably prevented the formation of clearly recognizable iPSC colonies (more than an 85% decrease), strongly suggesting that the observed effects of mdivi-1 on reprogramming efficiencies were due mostly to the inhibition of the process of reprogramming itself and not to a significant impairment of iPSC colony survival or growth. Indeed, when mdivi-1 treatment began on day 7, we found late changes in cell culture morphologies analogous to “background colonies” or “early colonies” that can be observed in untreated control cultures beginning on day 4 (Fig. 2B). The combined results obtained when MEFs were exposed to continuous or intermittent mdivi-1 clearly indicate that reprogramming of somatic cells into iPSCs is less efficient and slower in response to mdivi-1 inhibition of DRP1-mediated mitochondrial division.

## The DRP1-GTPase inhibitor mdivi-1 promotes the differentiation of established iPSCs

**Figure 3:**
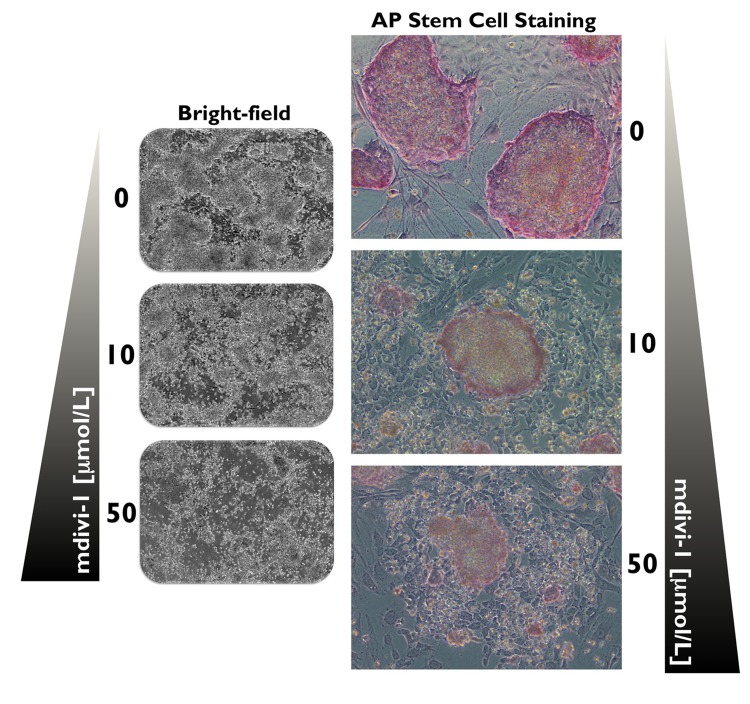
DRP1 activity is required for the maintenance of iPSCs Microphotographs show typical colony morphology of iPSCs with positive AP staining (red). In mdivi-1-treated cultures (5 days), normal undifferentiated phenotype with distinct iPSC colonies was not maintained. Remarkably, the colony morphology of the iPSCs was lost and differentiated cells with negative AP staining and some flattened fibroblast-like cells were formed after treatment with varying concentrations of the DRP1 inhibitor mdivi-1, as specified. Detection of AP activity, which is indicative of the non-differentiated state of iPSCs, was carried out using a commercial AP staining kit according to manufacturer's instructions.

Alkaline phosphatase (AP) is a universal pluripotency marker for all types of pluripotent stem cells, including embryonic stem cells, embryonic germ cells and iPSCs. Indeed, AP staining is widely used to identify emerging pluripotent colonies during the process of somatic reprogramming. We employed AP staining and observed the morphological changes of iPSCs to evaluate whether mitochondrial division is required for the maintenance of the undifferentiated state of iPSCs. At day 20 post-transduction, the established iPSCs were selected and passaged onto pre-seeded MEF feeder cells. The iPSCs were then exposed to two different concentrations of mdivi-1 for 5 days. Strong and uniform AP staining was detected in the untreated iPSC control colonies, demonstrating one of the properties attributed to pluripotent cells (Fig. 3). Remarkably, the typical morphology of established iPSC colonies with AP+ staining was significantly affected by mdivi-1 exposure. On the one hand, the colony morphology of the iPSC colonies (i.e., dense round cells with a well-defined edge) was lost, and they rather resembled fibroblast-like differentiated cells (Fig. 3). On the other hand, the AP staining was drastically reduced, which is indicative of differentiation. Thus, the prevention of mitochondrial division imposed by mdivi-1-inhibited DRP1 apparently led to differentiation and consequently disrupted the self-renewal of iPSCs.

## Mitochondrial fusion impedes somatic cell reprogramming to pluripotency: New insights into the role of mitophagy in cell stemness

Mitochondria certainly should play a role in the metabolic shift that enables somatic reprogramming to stemness because the physiology of mitochondria is inextricably linked to energy metabolism [42]. Specifically, mitochondrial structure and function have been suggested to be indicators of stem cell competence because low mitochondrial activity and relatively under-developed mitochondrial networks have been confirmed to be common features of stemness [43-48]. Vessoni and colleagues [49] hypothesized that autophagy could play an important role in mediating the remodeling of differentiated cells to a pluripotent state during the generation of iPSCs. Mitophagy would promote mitochondrial degradation during iPSC generation, allowing differentiated cells to reduce the amount of this organelle to ESC-like levels. To test a “metabolic state hypothesis” that links the mitochondrial state and cellular bioenergetics to the state of differentiation, Vessoni and colleagues [49] suggested that an increase in the number of developed mitochondria and the mitochondrial mass in iPSCs generated from autophagy-deficient cells (ATG7^−/−^) would argue for a pivotal role for autophagy during reprogramming. In the same way, the generation of iPSCs from differentiated cells might also be positively influenced by autophagy modulation. Because mitochondrial fission is a mediator of mitochondrial turnover (i.e., mitochondrial fission followed by selective fusion segregates dysfunctional mitochondria and permits their removal by autophagy) and because inhibiting mitochondrial fission results in the specific inhibition of mitochondrial autophagy before the phagophore is assembled [50, 51], we recently envisioned that pharmacological perturbation of mitochondrial dynamics before and after iPSC generation may illuminate mitophagy as a pivotal mechanism driving somatic reprogramming to stemness. Our current findings provide new insight into how mitochondria division is integrated into the reprogramming factor-driven transcriptional network that specifies the unique pluripotency of stem cells. Our data strongly suggest for the first time that the occurrence of mitophagy may be involved in the selective turnover of mitochondria prior to and during the reprogramming of somatic cells to iPSCs. In light of recent studies suggesting that changes in metabolism may play a role in enabling the reprogramming process to occur, instead of being a consequence of acquiring a pluripotent state, our data confirm a causal correlation between the bioenergetic state of somatic cells and their reprogramming efficiency. Future studies should elucidate whether the ability of mitophagy to directly shift the oxidative:glycolytic production ratios closer to those of pluripotent cells (i.e., somatic cells primarily utilize mitochondrial oxidation for proliferation, whereas pluripotent cells favor glycolysis) can molecularly explain the impact of mitochondria fusion/fission dynamics on the acquisition and maintenance of stem cell pluripotency.
